# PET Imaging of Epigenetic Influences on Alzheimer's Disease

**DOI:** 10.1155/2015/575078

**Published:** 2015-10-22

**Authors:** Paul J. Couto, Richard M. Millis

**Affiliations:** Department of Medical Physiology, American University of Antigua College of Medicine, St. John's, Antigua and Barbuda

## Abstract

The precise role of environment-gene interactions (epigenetics) in the development and progression of Alzheimer's disease (AD) is unclear. This review focuses on the premise that radiotracer-specific PET imaging allows clinicians to visualize epigenetically influenced events and that such imaging may provide new, valuable insights for preventing, diagnosing, and treating AD. Current understanding of the role of epigenetics in AD and the principles underlying the use of PET radiotracers for *in vivo* diagnosis are reviewed. The relative efficacies of various PET radiotracers for visualizing the epigenetic influences on AD and their use for diagnosis are discussed. For example, [^18^F]FAHA demonstrates sites of differential HDAC activity, [^18^F]FDG indirectly illuminates sites of neuronal hypomethylation, and the carbon-11 isotope-containing Pittsburgh compound B ([^11^C]PiB) images amyloid-beta plaque deposits. A definitive AD diagnosis is currently achievable only by postmortem histological observation of amyloid-beta plaques and tau neurofibrillary tangles. Therefore, reliable *in vivo* neuroimaging techniques could provide opportunities for early diagnosis and treatment of AD.

## 1. Introduction

In broad terms, Alzheimer's disease (AD) is a neurodegenerative disorder that leads to cognitive dysfunction. With a worldwide prevalence of 20 million, AD is the leading cause of dementia, associated with an increasingly aged global population [1]. Several overlapping hypotheses involving neurotransmitter dysregulation, oxidative stress, lipid metabolism, effects of metals (e.g., zinc) on metabolism, and effects of various genes have been purported to explain the causes of AD. One of the more outdated theories is the cholinergic hypothesis which suggests that forebrain acetylcholine deficiency is associated with destruction of the nucleus basalis, a main source of cholinergic neurons and the basis of anticholinesterasic drug treatments [2]. The cholinergic hypothesis is currently in disrepute largely because of inefficacy of cholinergic treatments. Nevertheless, a nicotinic acetylcholine receptor antibody is shown to play a role in neuroinflammation and accumulation of amyloid [3].

Categorically speaking, AD is part of a larger group of illnesses, designated amyloidosis, wherein there is accumulation of insoluble, spontaneously aggregating proteins known as amyloids. The amyloid hypothesis of AD suggests that AD results from dysregulation of the amyloid precursor protein (APP) gene on chromosome 21, analogous to the increase in brain amyloid in Down syndrome due to an extra copy of chromosome 21 (trisomy) [4]. The tau hypothesis of AD suggests that neurons are destroyed by neurofibrillary tangles of the microtubule components of the cytoskeleton, from hyperphosphorylation of the tau protein product of the microtubule-associated protein tau (MAPT) gene, also known to occur in neuroinflammation and other degenerative brain diseases [5, 6].

It was originally thought that a specific amyloid subtype, amyloid-beta (A*β*), accumulates in the brain from overproduction, but most scientists now agree that although autosomal dominant forms of AD result from overproduction, in most cases the amyloid build-up results from reduced A*β* clearance [7]. A*β* is an extracellular protein comprised of long, nonbranching, fibrillar protein assemblies of *β*-sheets. A*β* is derived from a larger peptide, APP. A*β* is formed from APP through abnormal cleavage by sequential action of proteases, *β*-secretase and *γ*-secretase [8]. The aggregation and accumulation of A*β* plaques in brain parenchyma is associated with functional changes in intricate cell signalling pathways which result in neuronal dysfunction and cognitive impairment. Reports stating that A*β* is neurotoxic are based mainly on observations that brain atrophy may be accelerated in immune therapies for AD by the mechanisms of increased A*β* solubility [9]; however, such neurotoxicity is not fully established by systematic research.

A signature feature of AD is the accumulation of intracellular neurofibrillary helical tangles comprised of tau protein. Healthy tau protein assists in the assembly of microtubule structures inside neurons. In AD, irregular posttranslational modifications result in hyperphosphorylation and insolubility, thereby rendering tau protein unable to interact with microtubules. A*β* deposits and tau tangles are required elements for the histopathological confirmation of an AD diagnosis [10]. Indeed, autopsy assessment indicating the presence of A*β* deposits and tau tangles is reported to be sufficient to account for the cognitive impairment in AD; however, the relative importance of the A*β* deposits versus the tau tangles is arguable [11, 12]. Other factors including, but not limited to, white matter lesions and related cerebral blood flow decrements [13], as well as vascular lesions involving blood-brain barrier breakdown [14], may contribute to the cognitive deficits observed in AD. The presence of apolipoprotein E4 (APOE4) is a known risk factor for AD, related to APOE4 interactions with A*β* in blood vessel walls that may decrease clearance of A*β* [15]. However, approximately one-third of APOE4 noncarriers with the clinical diagnosis of mild to moderate AD did not meet the positron emission tomography (PET) criteria for detecting A*β* plaque accumulation in the cerebral cortex but had sufficient amounts of neurofibrillary tangles to support the diagnosis of AD [16]. Although there is no clear evidence for other factors amplifying the degree of amyloid or tau pathology observed in AD brains, novel PET imaging radioactive tracers, to be described later in this review, are being employed to investigate this. The main purpose of this review is to raise the issue of how epigenetic mechanisms may play a role in PET imaging and in AD.

Comparison of measurements of amyloid deposits imaged with PET to white matter hyperintensities (WMH) observed with magnetic resonance imaging (MRI) suggests an important influence of small vessel cerebrovascular disease on AD [17]. Moreover, a role for synaptic proteins with amyloidogenic potential (SPWAP) is supported by the abnormal folding or aggregation of SPWAP that has been observed in the brains of AD patients [18]. Mutations can also give rise to A*β* plaques and hyperphosphorylated tau proteins, decreasing neural plasticity [19]. These findings suggest an extensive list of biochemical, physiological, and genetic factors which contribute to the development of AD.

There is growing evidence that epigenetic dysfunction plays a significant role in the development of AD. Abnormal epigenetic regulation of gene expression in the brain is a relatively new concept in neuroscience. Epigenetics encompasses a variety of externally mediated mechanisms which alter gene expression and phenotype. Epigenetic alterations do not involve direct changes in a gene's nucleotide sequences but, rather, change the transcriptional potential (or expression) and, therefore, the activity of the gene. These adjustments in gene expression are typically catalyzed by DNA methylases, histone acetyltransferases (HATs) and histone deacetylases (HDACs), and noncoding miRNAs, as well as by the processes of phosphorylation and ubiquitination [20]. Histone modifications and DNA methylation are the two major epigenetic dysregulations that appear to result in excess A*β* and neurofibrillary tangles of tau protein which contribute to neurodegeneration.

Currently, definitive diagnosis of AD is possible only by postmortem observation of A*β* plaques and tau neurofibrillary tangles histologically [21]. Therefore, it is in the best interests of an ever-increasing population of elderly patients with a presumptive diagnosis of AD to develop accurate, noninvasive* in vivo* diagnostic imaging techniques that will avoid the burdens of misdiagnosis and inappropriate treatments. To date, the most successful imaging technique for detecting and characterizing protein expression is the combined usage of molecule and enzyme-specific radiotracers with PET imaging. In comparison with MRI, PET is shown to possess a greater specificity and sensitivity for detecting proteins in small concentrations [21]. A PET radiotracer is a molecule to which a positron emission isotope, the most commonly studied and utilized in practice being fluorine-18 (^18^F), has been inserted [22]. PET neuroimaging radiotracers can be used to determine brain sites where epigenetic dysfunction has caused specific enzymes to be functional or nonfunctional. Various radiotracers with specificity for particular molecular substrates have been developed. The current radiotracers that are most useful for AD include [^18^F]FAHA, [^18^F]SAHA, [^64^Cu]CUDC, [^18^F]FDG, [^11^C]PiB, [^18^F]florbetapir, [^18^F]florbetaben, and [^18^F]flutemetamol. Pittsburgh compound B (PiB), [^18^F]florbetapir, [^18^F]florbetaben, and [^18^F]flutemetamol have been designed to directly bind and detect extracellular A*β* levels in the brain [23].

In this review, we summarize the mechanisms of epigenetic dysregulation seen in AD and outline the most effective imaging techniques for the visualization of the outcomes of such dysregulation. Early diagnosis, treatment, and, ultimately, prevention of AD may depend on how cognizant clinicians are of the radiotracers that can be used for PET imaging of the brain.

## 2. Epigenetics of Alzheimer's Disease and Related PET Imaging Methods

### 2.1. Histone Modification

**Figure 1:**
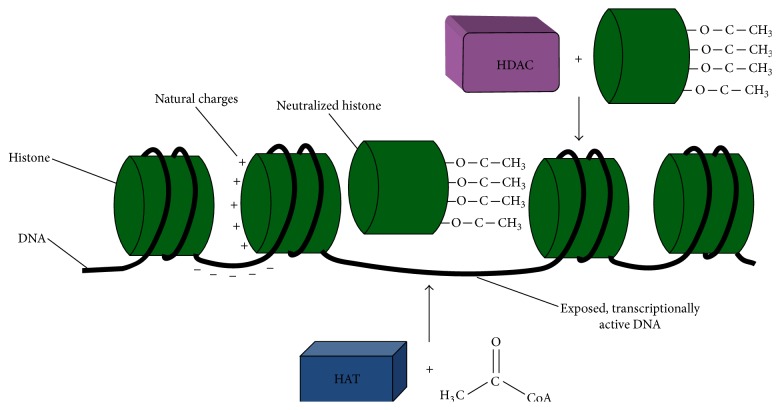
Histone regulation of gene expression. Positively charged histone octamers bind to the negatively charged phosphate backbone of DNA, condensing it into transcriptionally inactive heterochromatin. Histone acetyltransferase (HAT) enzymes donate acetyl groups from acetyl-coenzyme A to histone proteins, thereby neutralizing its positive charge, resulting in its dissociation from DNA, making it transcriptionally active. This relaxed form of chromatin, euchromatin, can then reassociate with DNA through the action of histone deacetylases (HDACs), which remove acetyl groups from the neutralized histone protein, returning it to transcriptionally inactive cationic heterochromatin.

Histones are positively charged octameric nuclear proteins that facilitate coiling of DNA into condensed chromatin. The negatively charged phosphate groups of DNA allow it to coil around the histone octamer, which is comprised of two pairs of H2a, H2b, H3, and H4 histone classes, forming a nucleosome, which is connected in succession by the linker histone H1. Histone acetyltransferases (HATs) and histone deacetylases (HDACs) regulate histone modifications which change the transcriptional potential and, therefore, the expression of genes, as depicted in Figure 1. Acetylation by HATs involves the transfer of negatively charged acetic acid groups from acetyl-coenzyme A to lysine residues on the amino-terminal tail of core histone proteins, which neutralizes the histone and dissociates it from the DNA, thereby exposing the DNA and increasing the transcriptional potential of the associated gene [24]. In contrast, HDACs catalyze deacetylation of histones, converting previously neutral histones back into their natural cationic form, permitting binding to the DNA, thereby decreasing the transcriptional potential of the particular gene and making it more resistant to degradation.

**Figure 2:**
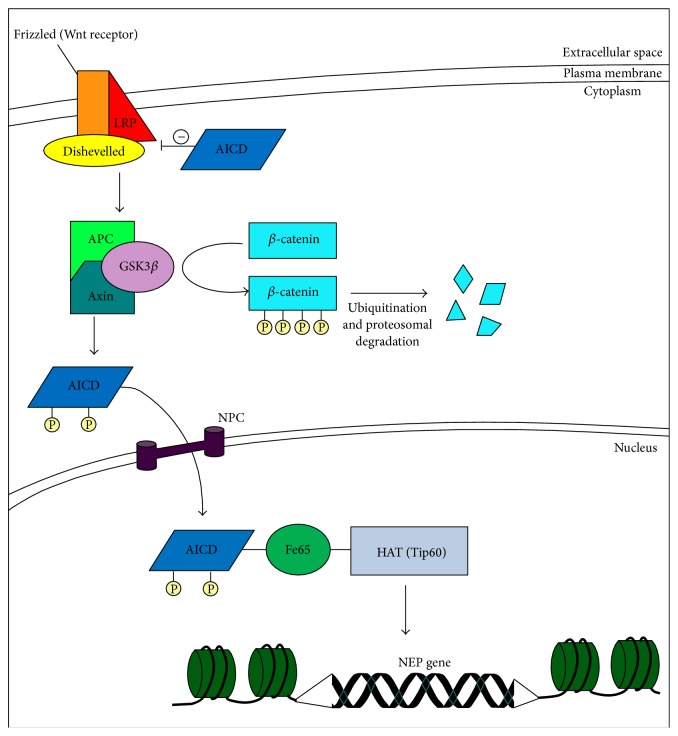
Wnt signaling and its relation to histone-regulated gene expression. In normal circumstances, when Wnt binds to its receptor, Frizzled, it blocks the degradation of cytosolic *β*-catenin, a transcription factor that then accumulates, translocates into the nucleus, and turns on transcription. LRP (low-density lipoprotein receptor-related protein) promotes Wnt-ligand binding to Frizzled. Dishevelled is a cytosolic protein associated with the tail of Frizzled. When AICD (amyloid precursor protein intracellular domain) is present, it inhibits Wnt signaling. The tumor suppressor protein APC (adenomatous polyposis coli) is then bound to the Axin protein, as well as to the kinase GSK3*β* (glycogen synthase kinase 3*β*), which triggers the phosphorylation of *β*-catenin that, in turn, triggers ubiquitination and degradation of *β*-catenin in the proteasome. The APC-Axin-GSK3*β* complex also phosphorylates AICD which translocates into the nucleus through a NPC (nuclear pore complex). Relevant to Alzheimer's disease, AICD binds to Fe65 (an A*β*-binding protein) which facilitates its binding to Tip60 (a type of HAT, histone acetyltransferase). This complex can then alter expression of the NEP (neprilysin) gene, which causes neuronal degradation.

In AD, there appears to be anomalous epigenetic regulation of histones, involving diverse cellular signaling pathways that impact overall brain activity. One such pathway involves the initial cleavage of APP by *β*- and *γ*-secretases, which generate A*β* plus an APP carboxy-terminal peptide, the APP intracellular domain (AICD) [8]. AICD strongly inhibits the Wnt signaling pathway and expression of the c-Myc gene, through its interaction with glycogen synthase kinase 3 beta GSK3*β*, as depicted in Figure 2 [8]. In the absence of Wnt-mediated signaling, GSK3*β* is known to complex with APC-Axin. In turn, the GSK3*β*-APC-Axin complex disables the *β*-catenin transcription activator phosphorylation mechanism, thereby enabling both its polyubiquitination and degradation in the proteasome [8]. Furthermore, AICD is able to translocate both into and out of the nucleus [25] and can interact with Tip60 (a type of HAT) directly, or indirectly through Fe65 (an A*β* binding protein) [26]. When AICD nuclear translocation and binding to Fe65 occur, AICD is phosphorylated by GSK3*β*, thereby increasing its neurotoxicity [27]. Once in the nucleus, AICD can modify the expression of the NEP (neprilysin) gene, which functions to terminate neuropeptide signals resulting in the neuronal degeneration associated with AD [28]. Another pathophysiologic feature of AD is irregular modification of DNA repair resulting from accumulation of phosphorylated histone H2AX in astrocytes, observed in regions of the hippocampus [25]. Correct DNA repair requires Tip60 acetylation of histone 4, and increased levels of histone H2AX affect the aforementioned AICD-Fe65-Tip60 interaction, which is necessary for association with chromatin and repair of DNA strand breaks [25]. Other histone modifications in AD involve hyperphosphorylation and hyperacetylation of histone 3 in hippocampal neurons, aberrant upregulation of histone 1 in astrocytes, and preferential binding of A*β* to linker histone H1 [24, 29].

### 2.2. Use of PET Radiotracers for Visualizing HDAC Activity

Noninvasive,* in vivo* neuroimaging techniques for visualizing the result of epigenetic dysregulations mostly involve chromatin-modifying enzymes, in particular HDAC activity. Development of probes for epigenetic regulatory enzymes like HDACs is complicated and significant technological obstacles need to be overcome for* in vivo* measurements to be uniformly reliable. Radiotracers must be small (<400 Da) and lipophilic to be able to cross the blood-brain barrier (BBB) [21]. They must also be distinguishable from other metabolites and resistant to metabolism/degradation in the bloodstream, have a high binding affinity that is specific for a substrate, and be able to produce a quantifiable PET signal [21]. Radiotracers must also have suitable nonspecific clearance rates so that they are rapidly washed out from areas without the substrate, thereby permitting sufficient contrast [30].

One such radiotracer that conforms to these requirements and allows for visualization of HDAC activity by PET is [^18^F]FAHA ([^18^F] 6-(fluoroacetamido)-1-hexanoicanilide) [31]. Inside neurons, [^18^F]FAHA is shown to be cleaved by class 2a HDAC enzyme subtypes (HDACs 4, 5, 7, and 9), thereby releasing [^18^F]FACE ([^18^F]fluoroacetate) which is metabolized further to [^18^F]fluorocitrate [31]. Because outward cellular transport of [^18^F]fluorocitrate is relatively slow due to its irreversible binding to aconitase, there is sufficient retention of radioactivity to permit visualization by PET and for determination of HDAC activity [31]. The findings presented by Yeh et al. [31] suggest that, despite its rapid metabolism to [^18^F]FACE, PET imaging with [^18^F]FAHA could be a viable approach for imaging brain HDAC activity and for developing novel class 2a HDAC inhibitors that have the potential to aid in understanding the role of HDACs in AD.

Alternatives to [^18^F]FAHA for the visualization of HDACs include [^18^F]SAHA ([^18^F]-suberoylanilide hydroxamic acid) [32] and [^64^Cu]CUDC-101 (7,4,3-ethynylphenylamino-7-methoxyquinazolin-6-yloxy-*N*-hydroxyheptanamide) [33]. [^18^F]SAHA is an analog of the most clinically relevant HDAC inhibitor, SAHA [32]. However, [^18^F]SAHA exhibits less BBB permeability than other available PET radiotracers, which limits its viability for the diagnosis of AD in comparison to [^18^F]FAHA [22, 32]. A nonfluorine-18-based radiotracer has also been developed which involves the copper-64 isotope, [^64^Cu]CUDC-101, the first of its kind [33]. [^64^Cu]CUDC-101 is reported to be specific for visualizing HDACs and appears to be a potentially useful tool for the discovery of new HDAC inhibitors and perhaps also for the diagnosis of AD [33]. Furthermore, its effective use of the copper-64 isotope also encourages further research into other PET radiotracers that may use the same isotope.

### 2.3. DNA Methylation

**Figure 3:**
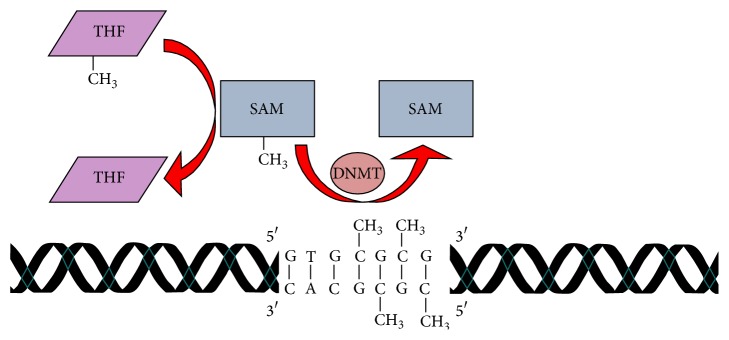
DNA methylation schematic. Addition of a methyl (CH_3_) group to cytosine residues on CpG islands effectively silences gene expression. Methylation of CpG islands is regulated by S-adenosylmethionine (SAM) and activity of DNA methyltransferase (DNMT). The methyl group derived from methylated tetrahydrofolate (THF) is donated to SAM and SAM then transfers the methyl group to cytosine nitrogenous bases in the DNA sequence. The ApoE (apolipoprotein E) gene (allele *ϵ*3, site A) is an example of where methylation silences (downregulates) ApoE and A*β* accumulates as a result of inhibiting A*β* processing [36].

Figure 3 shows that methylation of DNA is a mechanism for silencing genes, resulting in different baseline expression in different cells. Differential methylation is a reflection of cell functional specificity, mediated by differences in activity of the DNA methyltransferases (DNMTs) at CpG islands. CpG islands are gene sites where C-G nucleotides do not contain 5-methylcytosines, and where a methyl group can be transferred and transcription can be decreased. Methylation at CpG islands, therefore, decreases expression and silences a gene. DNA methylation can be increased (hypermethylation) or decreased (hypomethylation), potentially caused by epigenetic dysregulation from a wide variety of environmental influences, including improper nutrition [34]. Studies on monozygotic twins show decreased DNA methylation in the cerebral cortices of the twins diagnosed with AD in comparison to their unaffected siblings [34, 35]. This finding implies that differential epigenetic regulation of DNA methylation may be a predictor of susceptibility to AD. Using mass spectrometry in postmortem samples of brain tissues from AD patients, Wang et al. [36] demonstrated that there were several brain areas wherein DNA hypermethylation and gene silencing appeared to be a factor in the accumulation of A*β*. The APOE4 gene is an undisputed risk factor for late-onset AD (LOAD). APOE4 works with the presenilin-1 (PSEN1) gene on *γ*-secretase to process A*β*. APOE4 was found to be fully methylated at its 3′-CpG island and hypomethylated at its CpG-poor promoter region, suggesting that A*β* may accumulate from an imbalance in an epigenetic mechanism [36].

DNA methylation often occurs in conjunction with histone modifications. For DNA and histones to be methylated, the substrate S-adenosylmethionine (SAM) is required. The methyl group that is transferred to cytosine by DNMTs is derived from methylated tetrahydrofolate (THF) through its interactions with SAM [24]. In AD patients, SAM levels in brain and cerebrospinal fluid are decreased, leading to hypomethylation in metabolically significant brain sites [37]. For example, depletion of SAM is shown to directly result in hypomethylation of protein phosphatase-2A (PP2A), which, in turn, makes tau protein resistant to dephosphorylation [38]. This hyperphosphorylated tau protein accumulates and produces neurofibrillary tangles [38]. In this regard, SAM activity is shown to be epigenetically regulated by dietary alterations; dietary deficiency of folate and vitamin B12 is shown to inhibit DNA methylation [34]. Folate is an essential nutrient in diets and is required for the conversion of methionine to SAM [39]. The significance of dietary folate deficiency is demonstrated by the finding that low serum folate has been linked to atrophy of neurons in the hippocampus, associated with widespread hypomethylation of DNA [39]. Indeed, dietary folate sufficiency is purported to be an effective strategy for decreasing the risk for developing AD [34].

Biometals appear to play a role in epigenetic regulation of DNA methylation and amyloid deposition. Cherny et al. [40] have demonstrated that Zn(II) and Cu(II) stimulate the precipitation of A*β* into insoluble aggregates and occur in high concentration in cerebral cortical regions exhibiting amyloid plaque deposits. Additionally, exposure of infants to lead (Pb) during the early stages of brain development is reported to increase expression of APP, *β*-secretase, and the transcription regulator Sp1 [41]. In relation to methylation, an inverse relationship between Pb exposure and DNMT activity is shown to overproduce reactive oxygen species (ROS) and promote oxidative damage, associated with a predilection for AD [41]. Moreover, cadmium (Cd) exposure is reported to inhibit DNMT activity, associated with hypomethylation and increased production of both ROS and A*β* [42].

Some epigenetic mechanisms of DNA methylation involve interactions with micro-RNA (miRNA) [43]. miR-148a is a type of miRNA that is increased in AD and appears to decrease translation of mRNA encoding DNMT, resulting in hypomethylation of DNA [44]. Hypomethylation associated with decreased DNMT activity is reported to increase expression of nuclear factor-*κ*B (NF-*κ*B), a transcription regulator of proinflammatory cytokines and cyclooxygenase which catalyze production of prostaglandins and induce inflammation [45]. miRNAs also regulate Bcl-2, an antiapoptotic protein that is downregulated in AD, resulting in increased apoptosis and neurodegeneration [29]. However, miRNAs also affect DNA methylation indirectly, through A*β* itself. In that regard, miRNA subtypes miRs106 and miRs153 are shown to target mRNA encoding APP and modify the actions of *β*-secretase, thereby enhancing synthesis of A*β* [29].

### 2.4. PET Radiotracers for Visualizing Effects of DNA Methylation

Methylation typically represses gene expression. Hence, imaging the results of epigenetic processes affected by methylation involves identifying brain sites where gene expression is suppressed. Methylation-induced gene silencing is shown to be a mechanism for energy conservation during hypometabolic states in plants during drought [46] and in animals during cold stress [47]. Activity of the hypothalamic-pituitary-adrenal stress involved in producing the stress hormone cortisol is shown to be positively correlated with progression of AD [48]. High cortisol levels in hair are reported to occur with dysregulation of spindle and kinetochore-associated complex subunit 2 (SKA2) gene methylation, indicative of abnormal stress reactivity, purported to be similar to that in biological aging [49]. SKA2 methylation is associated with a decrement in thickness of the prefrontal cerebral cortex [50]. Cortical thickness decrements are also correlated with A*β* accumulation in PET brain scans of subjects selected from an Alzheimer's disease registry [51]. Taken together, these findings suggest the novel hypothesis that pathophysiological reactivity to stress, a known risk factor for AD, contributes to methylation-induced gene silencing that may be observed as brain tissue areas of hypometabolism in PET scans. The fact that hypometabolism could be a cause of A*β* amyloid deposition is consistent with the association of A*β* accumulation with the low regional cerebral blood flow and with the vascular lesions observed with A*β* in AD brain samples [13, 14].

The radiotracer most commonly used in practice for visualizing sites of hypometabolism is [^18^F]FDG ([^18^F]fluorodeoxyglucose), which concentrates radioactivity on areas with higher levels of glucose metabolism, thereby allowing for imaging brain regions of decreased metabolism [30]. Despite its widespread clinical usage, varying levels of diagnostic validity and reliability are reported for [^18^F]FDG-PET. Some state this radiotracer is reported to have 93%–95% accuracy for AD diagnosis [52, 53], while others raise several critical issues about using [^18^F]FDG-PET for diagnosing AD [21–23, 30]. Firstly, [^18^F]FDG analysis lacks clearly defined distinguishing features with ambiguous hypometabolism patterns that can be difficult to interpret qualitatively and image interpretation, therefore depending heavily on the training, experience, and skill of the observer [30]. Secondly, [^18^F]FDG-PET has low temporal resolution and is not specific for particular enzymes such as class 2a HDAC imaging with [^18^F]FAHA [21, 30].

### 2.5. PET Radiotracers for Visualizing Amyloid

**Figure 4:**
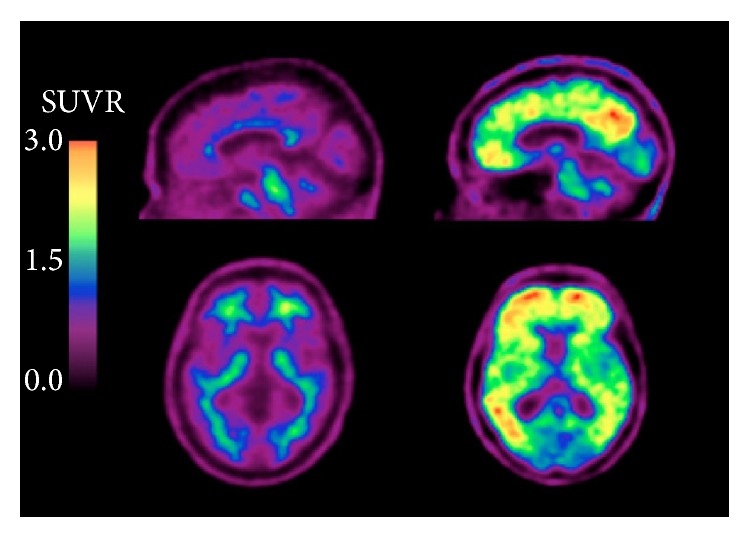
Sagittal and axial PET images using [^18^F]florbetaben. Both patients are female, are of the same age, and have the same MMSE score. The difference is that the patient on the left has mild cognitive impairment and demonstrates little retention of [^18^F]florbetaben, while the patient on the right has Alzheimer's disease and demonstrates high cortical [^18^F]florbetaben retention. This retention is represented in the standard pattern seen in AD: highest levels are retained in the posterior cingulate region as well as the frontal and temporal cortices ([59], reprinted with permission).

**Figure 5 fig5:**
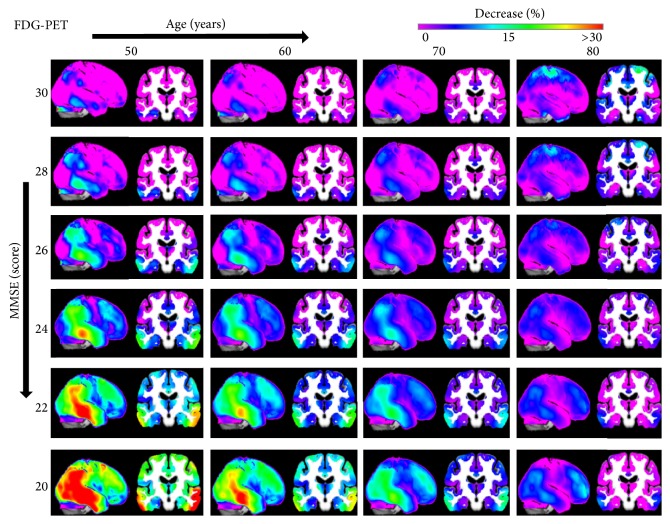
PET images using fluorodeoxyglucose (FDG) radiotracer. This model involves 3 variables: age, percent decrease in glucose metabolism, and Mini Mental State Examination (MMSE) scores in individuals diagnosed with AD using a healthy control group as baseline comparison (note that a MMSE score 27 or greater indicates normal cognition). In this study, the authors reported that an increase in AD severity is negatively correlated with grey matter volume, glucose metabolism, and MMSE scores. They also mention that the greater percent decrease in glucose metabolism in younger AD patients is both due to the dissociation between early- and late-onset AD and the effect of normal healthy aging in elderly patients resulting in a lesser percent decrease ([61], reprinted with permission).

**Figure 6 fig6:**
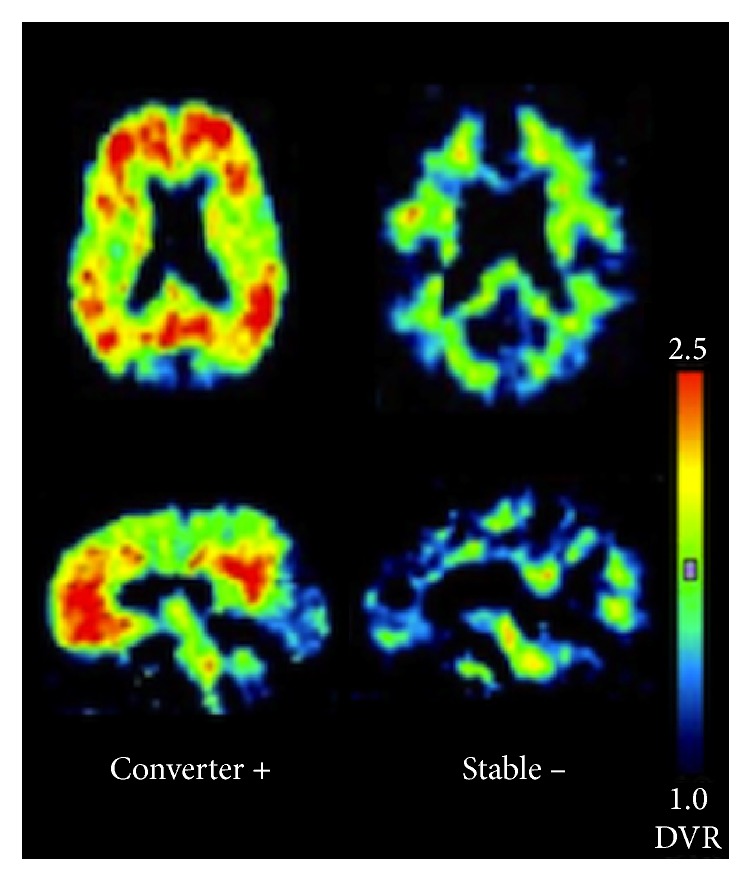
Transverse and sagittal PiB-PET scans from 2 patients with different degrees of amyloid-beta deposition. This image originates from a study examining patients with mild cognitive impairment (MCI) using positron emission tomography in combination with the radiotracer Pittsburgh compound B. It is noted that the PiB distribution volume ratio (DVR) of the patient that has progressed from MCI to AD (converter) is positive for amyloid-beta and shows a significant DVR, while the patient that is stable with MCI and has not progressed to AD (stable) is negative for A*β* and shows a lower DVR ([62], reprinted with permission).

**Figure 7:**
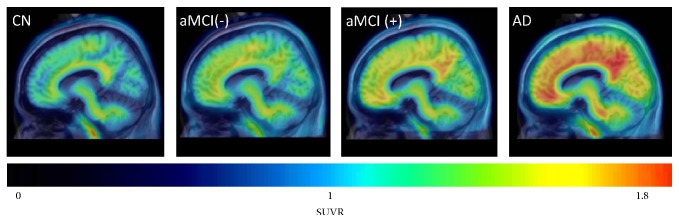
Representative sagittal PET images comparing the standard uptake value ratios (SUVRs) of [^18^F]florbetapir between cognitively normal (CN), amnestic mild cognitive impairment negative for cerebral A*β*, aMCI(−), amnestic mild cognitive impairment positive for cerebral A*β*, aMCI(+), and Alzheimer's disease (AD) patients. Note that higher [^18^F]florbetapir accumulation was especially prominent in parietal, temporal, and occipital gyri. This study also concluded that there was a significant difference between scores on the Mini Mental State Examination for neurocognitive testing and [^18^F]florbetapir SUVRs amid CN, aMCI(−), aMCI(+), and AD subjects ([56], reprinted with permission).

In one study, the effectiveness of [^18^F]FDG-PET for differentiating between AD and frontotemporal lobar degeneration (FTLD) was compared with a radiotracer that acts as a high-affinity ligand for A*β*, the carbon-11 isotope-containing Pittsburgh compound B (PiB) [23]. [^11^C]PiB-PET measures pathologic molecular features directly, while on the other hand [^18^F]FDG-PET measures the indirect effects of AD on brain structure. [^11^C]PiB-PET is reported to have greater sensitivity for diagnosing AD than [^18^F]FDG-PET, mainly by detecting the amyloid plaques [23]. Nevertheless, [^11^C]PiB-PET is not without its share of problems; its ^11^C isotope's short half-life of 20 min is reported to be a limitation compared to other radiotracers labeled with the ^18^F isotope, possessing a half-life of 110 min [23], but this has not been fully confirmed by research. A different radiotracer, [^18^F]florbetapir, seems to be a more useful technique for amyloid detection than [^11^C]PiB, with high diagnostic specificity for AD [54]. The longer radioactive half-life of florbetapir is an advantage because of its longer “shelf-life” while, in contrast, a significant amount of PiB's radioactivity would have been lost. Compared to an ^11^C isotope, the ^18^F isotope results in more decay events that can be detected, potentially enabling shorter imaging times and higher quality images. [^18^F]florbetapir also seems to measure similar levels of A*β* as postmortem histological analysis [54]. As a result, [^18^F]florbetapir was approved by the USFDA for AD imaging in 2012 [21]. [^18^F]florbetapir is shown to be effective for measuring the age-related increase in A*β* aggregates, as well as the differential distribution of A*β* across the cerebral cortices of aging individuals [55]. In Figure 7, Huang et al. [56] demonstrate that there could be cases found corresponding to different Braak amyloid stages [57]. Note that these [^18^F]florbetapir images do not represent a necessary progression of amyloid along the AD spectrum or an increasing level of amyloid moving from mild cognitive impairment to AD. Huang's amyloid-positive cases exhibit an amyloid burden in their mild cognitive impairment cases similar to AD, a finding that has been obtained in other studies [58]. Alternatives to [^18^F]florbetapir with similar half-lives and A*β* binding affinity include [^18^F]florbetaben and [^18^F]flutemetamol. [^18^F]florbetaben with PET has been shown to be a promising tool for early detection of AD pathology, illuminating areas with A*β* deposition in individuals with even mild cognitive impairment and allowing for earlier diagnosis [59]. Similarly, [^18^F]flutemetamol can demonstrate differential A*β* levels across various stages of neurodegeneration [60].  Table 1 lists the radiotracers which have been used for imaging epigenetic influences on AD and Figures 4 [Fig fig5] [Fig fig6]–7 present representative results of imaging using the radiotracers [^18^F]florbetaben [59], [^18^F]FDG [61], [^11^C]PiB [62], and [^18^F]florbetapir [56].

In addition, single-stranded RNA oligonucleotides (aptamers) have been synthesized to have high affinity and specificity for A*β* [65]. The rationale for using such radiotracers is based on the finding that polymeric A*β* plaques serve as a reservoir for monomeric A*β*. Monomeric A*β* is reported to bind an optically active, fluorescent RNA aptamer, *β*55, employed for near-infrared spectroscopic detection of A*β* with exquisite sensitivity and specificity [65]. This form of (monomeric) A*β* detected by a fluorescent aptamer probe is correlated with memory loss in rodent AD models [66]; however further research and development on ^18^F*β*55 and other RNA radiotracers for imaging human brain are required.

### 2.6. PET Radiotracers for Visualizing Tau Protein

It is noteworthy that a clinically effective PET radiotracer specific for the tau protein that can be used in practice has yet to be developed. A*β* deposits in extracellular space are shown to surround cells and, therefore, to make it difficult for tau ligands to pass into the intracellular space and bind with high specificity [64]. Likewise, the imaging of epigenetic activity by molecules akin to imaging the class 2a HDACs with [^18^F]FAHA is fraught with difficulty for diagnosing AD because of the ubiquity of such epigenetic molecules. The imaging of A*β* is confounded by its accumulation during neurodegeneration such as that associated with mild cognitive impairment [60]. However, there are a number of relatively specific tau-binding radiotracers, the distribution of which is shown to be quite different from those for detecting amyloid. Perhaps the most promising tau protein radiotracer is the aminophenyl quinolone [^18^F]THK which, along with three derivatives, is shown to have high specificity for tau protein tangles, in the absence of nonspecific binding to other cellular proteins [67]. However, some have concluded that [^18^F]THK-523's uptake pattern in AD patients was not significantly different from that of the controls, thereby rendering it inadequate for use [64]. In addition, nitropyridines seem to have binding affinity for the microtubule structural protein tubulin [68]. The nitropyridine radiotracer [^18^F]AV-1451 is purported to have potential in identifying AD because of high sensitivity for detecting tau protein in the form of tangles and of insensitivity to other forms of tau protein [63]. However, some nonspecific binding to hemorrhagic and melanin-containing structures has been reported for [^18^F]AV-1451 [63]. Thus, preliminary data show that [^18^F]AV-1451 has high selective binding affinity to tau protein aggregates, although it is accepted that further investigation into its clinical viability is still required [69, 70]. Lastly, hypermethylation of the dual-specificity phosphatase 22 (DUSP22) gene's promoter region has been found in AD hippocampal brain samples, and DUSP22 is known to play a key role in determining the phosphorylation status of microtubule-stabilizing tau proteins [71].

## 3. Conclusions

Noninvasive,* in vivo* PET imaging modalities are incredibly useful for the visualization of degeneration in AD. This degeneration has been shown by a wealth of research to be in part due to faulty epigenetic mechanisms. This overview of epigenetic factors in AD demonstrates how PET imaging with appropriate radiotracers can help in identifying the environment-gene interactions responsible for some of the specific pathophysiological features of this complex disease. Elucidating the molecular mechanisms involved in epigenetic dysregulation provides a micro-molecular approach to understanding why PET images of AD patients appear the way that they do. Cognizance of epigenetic-related changes may help motivate imaging practitioners to play a greater role in future, improved prevention and treatment strategies for AD. Ultimately, further development of PET radiotracers for the detection of features specific to AD is necessary, especially those targeted at tau neurofibrillary tangles. Translating current breakthroughs in PET radiotracer development into greater awareness among radiologists and other health care practitioners about the pathological features of AD may also improve earlier diagnosis of AD. Radiologists in particular have a great responsibility to be informed about the creation of novel prevention strategies for AD. Indeed, neuroimaging by PET appears to provide a tool for linking our current knowledge of epigenetic regulation with* in vivo* function in both normal and AD-affected brains.

## Figures and Tables

**Table 1:** PET radiotracers for visualizing epigenetic influences on Alzheimer's disease.

PET radiotracer	Measurement	Related epigenetic mechanism	Reference
[^18^F]FAHA	Class 2a HDAC	Histone modification, HDAC activity	Yeh et al., 2013 [31]

[^18^F]SAHA	Class 1 and 2b HDAC	Histone modification, HDAC activity	Hendricks et al., 2011 [32]

[^64^Cu]CUDC-101	HDAC	Histone modification, HDAC activity	Meng et al., 2013 [33]

[^18^F]FDG	Glucose	DNA methylation, metabolic activity	Mosconi et al., 2008 [52]; Dukart et al., 2013 [61]

[^11^C]PiB	Amyloid-beta	Molecular production of A*β*	Rabinovici et al., 2011 [23]; Hatashita and Yamasaki, 2013 [62]

[^18^F]florbetapir	Amyloid-beta	Molecular production of A*β*	Clark et al., 2011 [54]; Huang et al., 2013 [56]

[^18^F]florbetaben	Amyloid-beta	Molecular production of A*β*	Ong et al., 2013 [59]

[^18^F]flutemetamol	Amyloid-beta	Molecular production of A*β*	Thurfjell et al., 2012 [60]

[^18^F]AV-1451	Tau protein	Phosphorylation of tau protein	Marquie et al., 2015 [63]

[^18^F]THK	Tau protein	Phosphorylation of tau protein	Shah and Catafau, 2014 [64]
